# Addressing carbapenemase-producing extensively drug-resistant *Pseudomonas aeruginosa*: the potential of cefiderocol and ceftazidime/avibactam plus aztreonam therapy

**DOI:** 10.1007/s10096-025-05061-4

**Published:** 2025-02-18

**Authors:** María Milagro Montero, Sandra Domene-Ochoa, Núria Prim, Eliana Ferola, Carla López-Causapé, Marian Gomis-Font, Mario F. Ampuero-Morisaki, Daniel Echeverria, Luisa Sorlí, Sonia Luque, Eduardo Padilla, Santiago Grau, Antonio Oliver, Juan P. Horcajada

**Affiliations:** 1 Infectious Diseases Service, Hospital del Mar, Passeig Marítim 25-29, Barcelona, 08003 Spain; 2 Infectious Pathology and Antimicrobials Research Group (IPAR), Hospital del Mar Research Institute (IMIM), Barcelona, Spain; 3 Department of Medicine and Life Sciences (MELIS), Universitat Pompeu Fabra Barcelona, Barcelona, Spain; 4 Servicio de Microbiología y Unidad de Investigación, Hospital Son Espases, IdISBa, Palma de Mallorca, Spain; 5 Microbiology Service, Laboratori de Referència de Catalunya, Barcelona, Spain; 6 Pharmacy Service, Hospital del Mar, Barcelona, Spain; 7 CIBER of Infectious Diseases (CIBERINFEC CB21/13/00002 and CB21/13/00099), Institute of Health Carlos III, Madrid, Spain; 8 Microbiology service, Hospital Universitario Ramon y Cajal, Madrid, Spain

**Keywords:** *Pseudomonas aeruginosa*, Cefiderocol, Ceftazidime/avibactam, Aztreonam, PK/PD, Chemostat

## Abstract

This study evaluated the activity of cefiderocol and the combination of ceftazidime/avibactam (CZA) plus aztreonam against carbapenemase-producing extensively drug-resistant (XDR) *Pseudomonas aeruginosa* isolates. Nine clinical XDR *P. aeruginosa* isolates with different sequence types and class A (GES) or B (VIM, IMP or NDM) carbapenemases were analysed. Time-kill assays assessed bacterial load reduction for each treatment, while chemostat experiments on four isolates validated these findings. All isolates showed resistance to CZA, with four also resistant to aztreonam. Seven isolates were susceptible to cefiderocol, but two displayed borderline susceptibility (MIC 2–4 mg/L). Time-kill assays demonstrated bactericidal activity by cefiderocol in six isolates at 24 h, while CZA plus aztreonam showed bactericidal effects in three isolates and synergistic/additive effects in four isolates. In the chemostat model, cefiderocol and CZA plus aztreonam were bactericidal in all four tested isolates, with cefiderocol showing greater bacterial reduction in three of these isolates. Both cefiderocol and CZA plus aztreonam achieved significant reductions in bacterial counts compared to controls, but there was no significant difference between cefiderocol monotherapy and the combination. Both cefiderocol and CZA plus aztreonam demonstrated activity against XDR *P. aeruginosa* carrying metallo-β-lactamase (MBL) and/or serine-β-lactamase (SBL) carbapenemases. Cefiderocol was the only consistently effective monotherapy with a bactericidal effect across all tested isolates in the chemostat model.

**Supplementary Information:**

The online version contains supplementary material available at 10.1007/s10096-025-05061-4.

## Introduction

Antimicrobial resistance is a critical global health issue, driven by antibiotic misuse and the exceptional adaptability of certain pathogens. Among these, *Pseudomonas aeruginosa* stands out as a major cause of nosocomial and healthcare-associated infections, especially in patients with compromised immune systems [1, 2, 3, 4]. This pathogen rapidly acquires resistance genes, evolving into multidrug-resistant (MDR) and extensively drug-resistant (XDR) strains. In particular, carbapenemase-producing XDR *P. aeruginosa* isolates pose significant challenges, as they are linked to higher morbidity and mortality rates compared to infections with susceptible strains [5, 6, 7, 8].

The rapid dissemination of carbepenem-resistant *P. aeruginosa* strains, particularly those producing serine (SBL) and metallo-β-lactamases (MBL), severely limits treatment options [9]. The most common prevalent carbapenemases in *P. aeruginosa* include class A (GES and KPC) and MBLs (VIM, NDM and IMP) [10], which compromise the efficacy of multiple antibiotic classes. Given the limited effectiveness of existing treatments and the potential for further resistance, new therapeutic approaches and agents are critically needed.

Aztreonam, an β-lactam antibiotic, is not hydrolysed by MBLs; however, it is vulnerable to breakdown by SBLs, such as AmpC and ESBLs, commonly found in *P. aeruginosa* [11]. Ceftazidime/avibactam (CZA), a β-lactam/β-lactamase inhibitor combination, has shown promise due to its broad coverage against XDR pathogens, but it is ineffective against MBL-producing bacteria [12, 13, 14]. Combining CZA with aztreonam has emerged as a promising approach to restore bactericidal activity against MBL-producing isolates by inhibiting other β-lactamases that hydrolyze aztreonam [15].

Recent research on cefiderocol, a novel siderophore cephalosporin, has highlighted its potent in vitro activity against Gram-negative bacteria, including XDR *P. aeruginosa* [16, 17, 18]. Cefiderocol penetrates cells by utilizing both porins and iron transport systems, providing stability against a wide range of β-lactamases, including MBLs [19]. However, further investigation is required to determine its efficacy in combination with other therapies or as monotherapy against carbapenemase-producing strains.

The present study aimed to evaluate the in vitro efficacy of cefiderocol and the combination of CZA plus aztreonam against a diverse set of clinical XDR *P. aeruginosa* isolates carrying both SBL and MBL carbapenemases. By leveraging pharmacokinetics/pharmacodynamics (PK/PD) models such as time-kill and chemostat assays, we aim to assess the potential bactericidal and synergistic effects of these treatments, providing insight into therapeutic strategies for highly resistant infections.

## Materials and methods

### Bacterial isolates and susceptibility testing

Nine XDR *P. aeruginosa* clinical isolates were obtained from different Spanish hospitals in the context of two multicentric studies performed in 2017 and 2019 [21, 22]. These isolates were previously characterized by whole genome sequencing, and the main mechanisms of antimicrobial resistance were analysed. They were preserved in Muller-Hinton broth supplemented with 20% glycerol and stored at -80ºC.

Antimicrobial susceptibility testing was performed and interpreted according to EUCAST breakpoints (V13.1) and guidelines for broth microdilution using cation-adjusted Muller-Hinton broth (CAMHB, Becton-Dickinson and Co) for CZA, aztreonam and aztreonam/avibactam, and iron-depleted cation-adjusted Muller-Hinton broth (ID-CAMHB) for cefiderocol [23]. The reference strain PAO1 was included in the susceptibility testing experiments as quality control.

### Culture media

CAMHB was prepared according to the manufacturer’s instructions. Tryptic Soy Agar II (TSA II, Becton-Dickinson and Co.) used for colony quantification, was prepared following the manufacturer’s instructions. Columbia blood agar plates (COS) (bioMérieux) were used to reculture the isolates.

For cefiderocol testing, iron-depleted liquid media was required. ID-CAMHB was prepared following the CLSI recommendations, finally adjusted to pH 7.3 using hydrochloric acid and kept at 4ºC until usage [24]. To ensured proper preparation of the ID-CAMHB medium, a minimum inhibitory concentration (MIC) of cefiderocol was tested on the reference control strain (PAO1).

### Antibiotics

Aztreonam and ceftazidime were from Merck & Co., Inc (Kenilworth, NJ) and avibactam was provided by Pfizer (Ringaskiddy, County Cork, Ireland). Stocks were prepared according to the European Committee on Antimicrobial Susceptibility Testing (EUCAST) recommendations [23].

Clinical vials of cefiderocol (Fetcroja^®^, Shionogi, Inc) were used. The powder (1 g) was reconstituted with 10 mL of water for injection.

Concentrations for time-kill experiments were based on area under the curve (AUC) serum levels for 24 h (h): for aztreonam 2 g every 6 h, AUC_24_ of 1050 µg · h/ml [25]; for ceftazidime 2 g every 8 h, AUC_24_ of 800 µg · h/ml [26]; for avibactam 2 g every 8 h, AUC_24_ of 147 µg · h/ml [26]; and for cefiderocol 2 g every 8 h, AUC_24_ of 1050 µg · h/ml [27].

In the chemostat model, antibiotics were administered to simulate free plasma concentrations in critically ill patients under treatment for several infections. The simulated CZA dosing regimen was 2/0.5 g every 8 h by intravenous infusion over 2 h (current standard) to achieve a free maximum concentration of 74 mg/L (avibactam fixed at 4 mg/L), with a simulated elimination half-life of 2 h [17]. The simulated aztreonam dosing regimen was 2 g every 6 h by intravenous infusion over 1 h to achieve a free maximum concentration of 110 mg/L, with a simulated elimination half-life of 2 h [17]. For cefiderocol, the simulated dosing regimen was 2 g every 8 h by intravenous infusion over 3 h, to achieve a free maximum concentration of 142 mg/L and with a simulated half-life of 2 h [27].

### Time-kill curves

Time-kill curves were conducted in duplicate for each isolate and each experimental condition. Time-kill experiments were performed with each antibiotic alone and in combination at clinically achievable free drug concentrations.

Bacterial isolates were streaked onto Sheep Blood Agar plates (bioMérieux) and further incubated aerobically at 37 °C for 18–24 h. The overnight culture of each isolate was diluted in 30 mL of CA-MHB and incubated at 37 °C in a water batch shaker for 1–2 h to achieve early-log-phase growth. The inoculum used to start the time-kill assay was calculated by determining the optical density (OD) at 630 nm using a spectrophotometer. Erlenmeyer flasks were used for each isolate and time-kill experiment, each containing 30 mL of CAMHB supplemented with the corresponding antibiotics. The final bacterial inoculum was approximately 6 to 7 log_10_ CFU/mL per flask. Flasks were incubated at 37ºC in a shaker water bath for 24 h. Bacterial growth was measured at different time points (0, 4, 8, and 24 h). A 1-mL aliquot was obtained from each flask at each time point and centrifuged at 13,000 rpm for 3 min. Supernatants were discarded, and bacterial pellets were washed with sterile saline solution to minimize drug carryover. Serial decimal dilutions of samples were performed in CAMHB. 50 µL of each dilution were streaked onto TSA agar plates. Plates were incubated at 37ºC for 18 to 24 h. Colony-forming units (CFU) were quantified after overnight incubation; the bacterial density from the original sample was obtained considering the dilution factor. The limit of quantification was stablished at 400 CFU/mL (equivalent to 20 colonies per plate). All data were converted to a log_10_ scale.

### Pharmacodynamic parameters

Bactericidal effect was defined as a decrease of ≥ 3log_10_ CFU/mL in the colony count after 24 h, starting from the initial bacterial density. Synergy was defined as a reduction of at least ≥ 2log_10_ CFU/mL in the colony count after 24 h when comparing the antibiotic combination with the most potent individual drug. Additive effect was defined as a reduction of ≥ 1-<2log_10_ CFU/mL in the colony count after 24 h when comparing the combination with the most active antibiotic alone. In the present time-kill studies, a ΔCFU/mL ≥ 2.80log_10_ was considered bactericidal. The same pharmacodynamic parameters were used for the chemostat results [28].

### Chemostat model

Four XDR *P. aeruginosa* isolates belonging to the most common high-risk clones were selected due to their high prevalence in Spain [5]. A one-compartment in vitro chemostat model was performed to validate the activity of the antibiotics studied by time-kill curves. Chemostat experiments were conducted in duplicate for each isolate and each experimental condition. The chemostat model consisted of four independent glassware reactors. Isolates were cultured on COS plates (Columbia Agar with 5% Sheep Blood) and incubated at 37ºC aerobically overnight. Colonies were inoculated into an Erlenmeyer flask containing 35 mL of CAMHB and incubated at 37ºC overnight in a water bath shaker. Four Reactors were filled with 100 mL of CAMHB and one was filled with 100 mL of ID-CAMHB. Reactors were filled with 100 mL of CAMHB and were supplemented with the corresponding concentration of the selected antibiotic. One reactor was filled with 100 mL of ID-CAMHB and supplemented with cefiderocol. All antibiotics were infused via antibiotic pumps (CADD-Legacy^®^ PLUS; Smiths Medical ASD, Inc.). A reactor without antibiotic was included as a control. The chemostat experiment was placed in an incubator at 37ºC for 48 h. Fresh broth was supplied via a peristaltic pump (Masterflex L/S model 7524-40; Cole-Parmer Instrument Company, Vernon Hills, IL) programmed to achieve the human-simulated half-life of the antimicrobial being tested. Samples were obtained from each of the reactors at specific time points (0, 2, 4, 8 and 24 h) and were centrifuged at 13,000 rpm for 3 min. Serial dilutions in CAMHB were performed with each bacterial suspension. 50 µL of these diluted samples were plated onto TSA II plates and incubated at 37 °C overnight. Colonies were counted after the incubation, and CFU/mL were determined for each condition.

### Pharmacokinetic studies

Antibiotic concentrations were collected from the chemostat studies at predetermined time points until the end of the study, and immediately stored at -80ºC until analysis. These samples were taken to validate the antibiotic concentrations previously modelled. Pharmacokinetic samples were determined by high-performance liquid chromatography (HPLC) and validated by a simple linear regression model (*r*^*2*^*)*.

### Statistical analysis

In the chemostat model, differences in bacterial concentrations (in logarithmic phase) among antibiotic regimens for each included isolate were assessed using analysis of variance (ANOVA). To ensure the appropriateness of ANOVA application, data normality was verified via the Shapiro-Wilk test, and variance homogeneity was evaluated using Levene’s test. Upon demonstration of statistically significant differences between antibiotic regimens within an isolate by ANOVA, a post-hoc analysis employing Tukey’s test was conducted to delineate these differences. The means of differences (x 𝚫), their respective 95% confidence intervals (CI) and the adjusted p-values for multiple comparisons were reported. A p-value < 0.05 was considered statistically significant. The analysis was performed using the R programming language in the RStudio software version 2023.12.1.

## Results

### In vitro antimicrobial susceptibility studies

The antibiotic susceptibility profiles and previously characterized antibiotic resistance mechanisms of the nine XDR *P. aeruginosa* isolates are shown in Table 1. All isolates were resistant to CZA (MIC > 8/4 mg/L). Four isolates were resistant to aztreonam (MIC > 16 mg/L). Aztreonam/avibactam MICs were similar to those of aztreonam alone, except for the GES-1-producing isolate. Seven isolates were susceptible to cefiderocol (MIC ≤ 2 mg/L), and two showed borderline susceptibility with MIC values between 2 and 4 mg/L.

**Table 1:** Characterization of resistance mechanisms and minimal inhibitory concentrations (mg/L) of the antibiotics tested for the used panel of *Pseudomonas aeruginosa* isolates

Isolate	Sequence type	β- lactamases	Mutations in AmpC regulators^b^	Mutations in MexAB-OprM regulators^b^	AZT MIC (*R* > 16 mg/L)^a^	AZT/AVI MIC^a^	CZA MIC (*R* > 8 mg/L)^a^	FDC MIC (*R* > 2 mg/L)^a^
10 − 009	ST111	VIM-2	*ampD*-Q88L	-	> 64	> 64	> 32	0.5
07–016	ST175	GES-5	-	-	8	8–16	32	≤ 0.03
12–012	ST175	VIM-20, OXA-2	-	-	8	8	32	0.06
06–042	ST235	VIM-47	-	-	32	16–32	32	0.5
01–008	ST253	VIM-1	-	-	4	4	> 32	2–4
CLE02-006	ST664	IMP-1	-	*nalD*-nt398Δ2	32	16–32	> 32	2
MUR01-018	ST111	IMP-33	-	-	16	16	> 32	2–4
MAD02-009	ST235	GES-1, GES-5, OXA-2	-	-	>64	32	> 32	0.5
PA04004	ST654	NDM-1, GES-5	-	-	8	8–16	> 32	0.12

^a^Abbreviations: MIC, minimum inhibitory concentration; AZT, aztreonam; AVI, avibactam; CZA, ceftazidime/avibactam; FDC, cefiderocol

^b^Mutations confirmed to be involved in *ampC* or *mexAB-oprM* overexpression through real time RT-PCR

Four isolates were further analysed using the chemostat model. These isolates were selected based on their representation of key resistance mechanisms, clinical relevance, association with high-risk clones, and alignment with the study objectives, ensuring a robust and clinically meaningful evaluation of the therapies. All isolates were resistant to CZA, two were resistant to aztreonam, and all were susceptible to cefiderocol. The isolates harboured carbapenemases belonging to Ambler class A or B, with one also exhibiting AmpC hyperproduction.

### Time-kill experiments

Time-kill curves were performed to evaluate the effects of aztreonam, CZA, cefiderocol, and the combination of CZA plus aztreonam against nine XDR *P. aeruginosa* isolates (as shown in Online Resource). Time-kill results showing the bactericidal and synergistic/additive effects of each antibiotic regimen against *P. aeruginosa* isolates are shown in Table 2.

**Table 2:** Results of time-kill curves of each antibiotic regimen against *Pseudomonas aeruginosa* isolates

Antibiotic regimen	% of isolates		
	Bact 8 h	Bact 24 h	Add/Syn 24 h
CZA + AZT	89%	22%	44%
Cefiderocol	100%	67%	NA

^*^Abbreviations: AZT, aztreonam; CZA, ceftazidime/avibactam; Bact, bactericidal effect; Add/Syn, Additive/Synergistic effect. NA: not applicable

Bacterial growth without antibiotic reached 10^8^-10^9^ CFU/mL at 24 h across all isolates, representing the baseline growth. Cefiderocol monotherapy achieved the highest bacterial reduction, with a decrease of 3–5 log_10_ CFU/mL, in all isolates except two, belonging to the ST253 (01–008) and ST654 (PA04-004) clones. Against these two isolates, cefiderocol and the combined therapy of CZA plus aztreonam had the same activity. Cefiderocol had bactericidal effect against six isolates (ST111 [10 − 009], ST235 [MAD02-009], ST175 [12–012], ST111 [MUR01-018], ST664 [CLE002-006] and ST654 [PA04-004]). The combination of CZA plus aztreonam showed a bactericidal effect against isolates ST111 (MUR01-018) and ST654 (PA04-004). This combination demonstrated a synergistic effect in ST235 (MAD02-009) and an additive effect in isolates ST654 (PA04-004), ST111 (10 − 009) and ST664 (CLE002-006). No bactericidal effect was achieved using CZA and aztreonam. monotherapies.

The time-kill experiments were also evaluated at 8 h. All isolates showed a decrease of 3–5 log_10_ CFU/mL when treated with cefiderocol or the combination of CZA plus aztreonam. Cefiderocol was bactericidal against all isolates at 8 h. Monotherapies with CZA and aztreonam caused a reduction of 2–4 log_10_ CFU/mL in the bacterial load of five and nine isolates, respectively. The combination of CZA plus aztreonam was bactericidal against all isolates except one (ST235 [06–042]). An additive effect with this combination was observed in one isolate (ST654 [PA04004]).

### Chemostat experiments

**Fig. 1:**
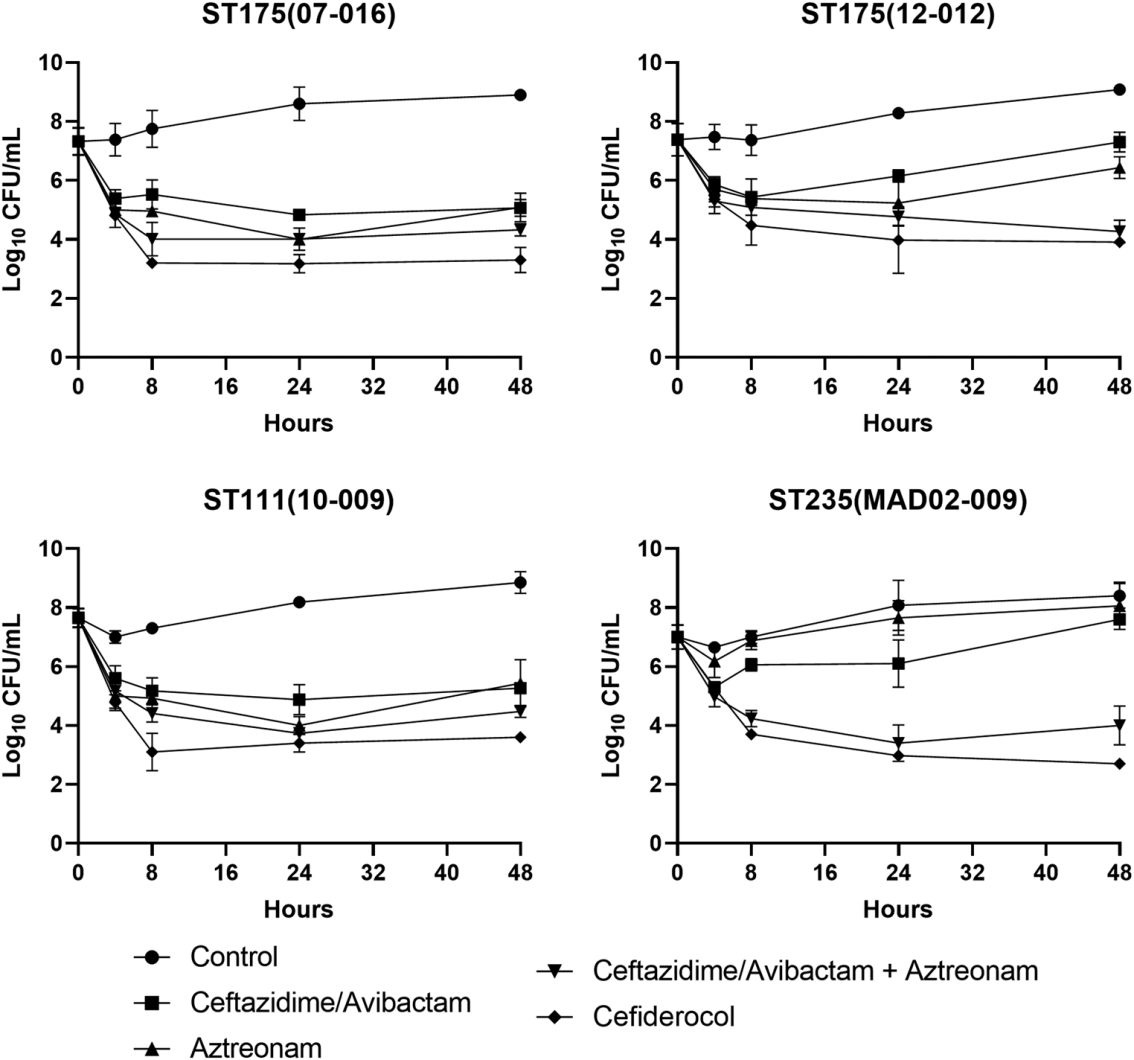
In vitro chemostat model against *Pseudomonas aeruginosa* isolates. Values shown are mean numbers of CFU/mL over 48 h for each antibiotic

The chemostat model served to validate findings from the time-kill assays, providing a dynamic PK/PD simulation over 48 h. The administration of cefiderocol and the combination of CZA plus aztreonam was evaluated. Monotherapies of CZA and aztreonam were also studied. The results of chemostat model are shown in Fig. 1.

Cefiderocol was the most active monotherapy, achieving greater bacterial reductions than other monotherapies across all isolates. Both cefiderocol and the combined therapy of CZA plus aztreonam achieved a bactericidal effect in the all isolates (Table 3). In the chemostat model, cefiderocol and CZA plus aztreonam achieved a reduction of 3–4 log CFU/mL. The combination of CZA plus aztreonam showed a synergistic effect against two of the four isolates, but did not demonstrate synergy in the remaining two isolates, where one of the monotherapies achieved a similar bacterial reduction as the combination.

**Table 3:** Comparison of bactericidal effects among antibiotic regimens in the four *Pseudomonas aeruginosa* isolates included in the chemostat model

Antibiotic regimen	Isolate			
	ST111 (10 − 009)	ST175 (07–016)	ST175 (12–012)	ST235 (MAD 02–009)
AZT vs. Control	-2-21	-2.24	-0.95	1.09
CZA vs. Control	-2.38	-2.26	-0.09	0.6
CZA + AZT vs. Control	**-3.17**	**-3**	**-3.12**	**-3**
Cefiderocol vs. Control	**-4.05**	**-4.03**	**-3.48**	**-4.3**

^*^Abbreviations: AZT, aztreonam; CZA, ceftazidime/avibactam

*Bactericidal activity (≥ 3 log10 CFU/mL reduction) is highlighted in bold

Regarding monotherapies, neither CZA nor aztreonam alone achieved a bactericidal effect in any isolate. Final regrowth was observed for the ST175 (12–012) with both monotherapies, and for the ST111(10 − 009) with the aztreonam monotherapy. For ST111(10 − 009), the bacterial load in CZA monotherapy resembled the control without antibiotic.

The variance analysis (ANOVA) was conducted to compare bacterial concentrations across different antibiotic regimens for each isolated, showing statistically significant differences (*p* < 0.05). Consequently, a post-hoc analysis using the Tukey test was performed to accurately identify these specific differences, which are summarized in Table 4. For all isolates, the combination of CZA plus aztreonam and cefiderocol monotherapy showed significant reductions in bacterial concentration compared to the control group. However, no statistically significant differences were found between the combination therapy of CZA plus aztreonam and cefiderocol monotherapy in any of the isolates.

**Table 4:** Comparative analysis of bacterial concentrations among antibiotic regimens of isolates included in the chemostat model

Isolate												
Antibiotic regimen	ST111 (10 − 009)			ST175 (07–016)			ST175 (12–012)			ST235 (MAD02-009)		
	x 𝚫^a^	CI 95%	*p* ^b^	x 𝚫^a^	CI 95%	*p* ^b^	x 𝚫^a^	CI 95%	*p* ^b^	x 𝚫^a^	CI 95%	*p* ^b^
AZT vs. Control	-2.39	-4.99 -0.2	0.08	-2.72	-5.14 -0.3	0.024	-1.89	-3.9 0.12	0.071	-0.27	-2.52 1.98	0.996
CZA vs. Control	-2.08	-4.68 0.52	0.157	-2.37	-4.78 0.06	0.06	-1.5	-3.5 0.52	0.211	-1.01	-3.27 1.24	0.666
CZA + AZT vs. Control	-2.71	-5.30 -0.11	0.039	-3.1	-5.51 -0.67	0.009	-2.56	-4.57 -0.55	0.009	-2.7	-4.95 -0.45	0.014
Cefiderocol vs. Control	-3.29	-5.89 -0.69	0.009	-3.63	-6.05 -1.21	0.002	-2.9	-4.91 -0.89	0.003	-3.1	-5.35 -0.84	0.004
Cefiderocol vs. CZA + AZT	-0.59	-3.13 2.01	0.96	-0.54	-2.96 1.88	0.96	-0.34	-2.35 1.67	0.986	-0.39	-2.64 1.86	0.98

^a^ Mean of differences in bacterial concentration (in logarithmic phase) between antibiotic regimens

^b^ Tukey-adjusted p-value. Statistically significant differences are highlighted

^*^Abbreviations: AZT, aztreonam; CZA, ceftazidime/avibactam

### Antibiotic exposures

**Fig. 2:**
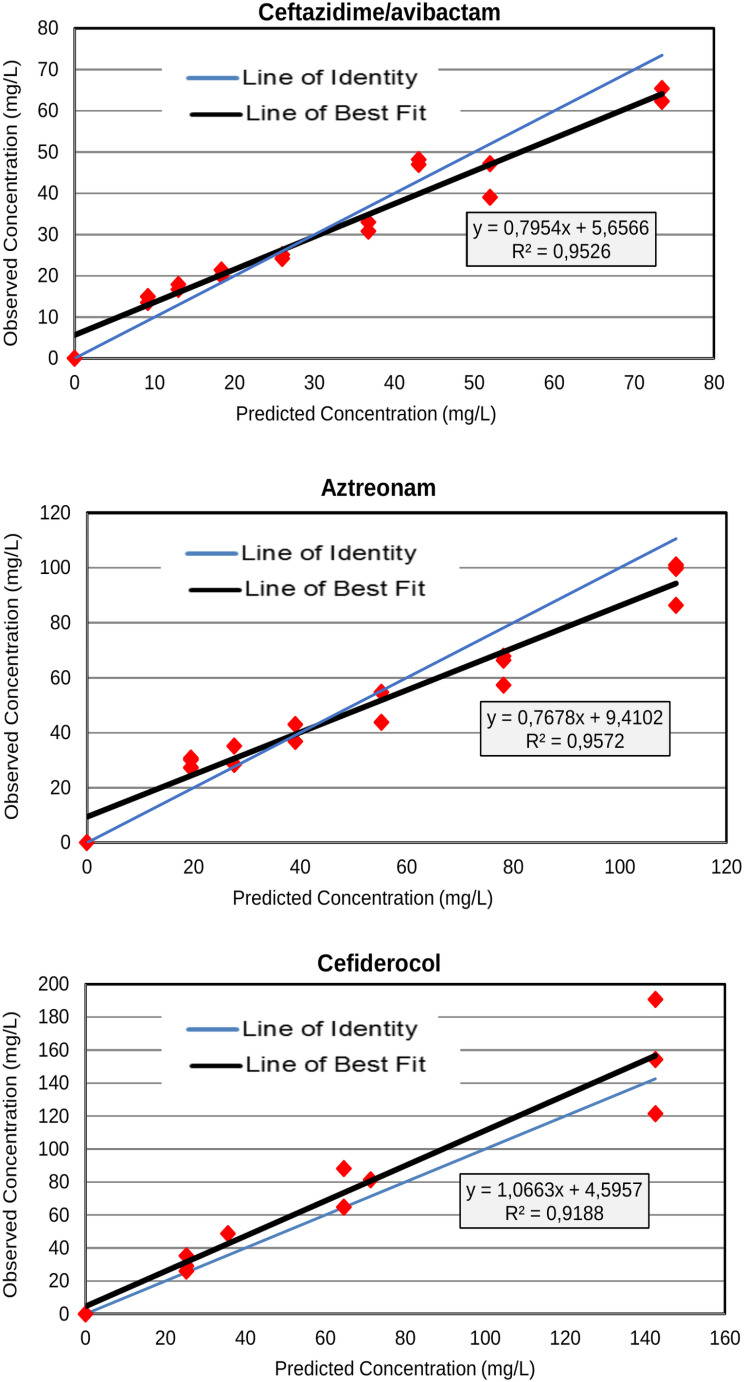
Relationship between observed and targeted antibiotic concentrations over the 48 h of the chemostat experiments

The observed concentrations and pharmacokinetic parameters calculated for all antibiotic regimens over the 48 h of the chemostat experiments are shown in Fig. 2. The observed versus predicted drug exposures were satisfactory for all regimens, with *r*^*2*^ values exceeding 0.90.

## Discussion

Opportunistic infections caused by XDR *P. aeruginosa* have become a significant public health concern due to limited treatment options. The combination of avibactam with aztreonam has been shown to restore the activity of the aztreonam against MBL- and SBL- producing Gram-negative pathogens by inhibiting the co-carriage of non-MBL β-lactamases such as ESBLs and AmpC enzymes [29]. Additionally, previous in vitro studies have demonstrated that the combination of CZA plus aztreonam achieves a bactericidal effect against XDR *P. aeruginosa* [17]. Cefiderocol, the most recent addition to the limited therapeutic arsenal for extensively drug-resistant Gram-negative bacilli, has exhibited superior activity in vitro when compared to other therapies [30, 31]. In vivo studies have also supported the use of cefiderocol against carbapenemase-producing Gram-negative bacilli, including XDR *P. aeruginosa* isolates [32, 33, 34, 35]. In this context, our study aimed to analyse the effect of cefiderocol and the combination of CZA plus aztreonam on XDR *P. aeruginosa* isolates producing various carbapenemases to identify the most effective therapy.

Time-kill experiments were conducted on a collection of XDR *P. aeruginosa* isolates with diverse β-lactam resistance mechanisms. These isolates varied in their susceptibility to aztreonam, although all were resistant to CZA. All isolates were susceptible to cefiderocol, but two could not be strictly categorized as susceptible according to EUCAST criteria. This uncertainty stemmed from cefiderocol MIC values repeatedly ranging between 2 and 4 mg/L in these isolates, likely due to strain-dependent factors and technical variability in susceptibility testing [24]. While this study focused on the phenotypic evaluation of cefiderocol activity, specific genetic determinants, such as mutations in iron transport genes (*fepA*,* cirA*,* piuA*,* pvdE*) or efflux pump overexpression, could influence susceptibility [19, 20]. These genetic factors were not systematically evaluated but warrant further investigation to better understand variability in cefiderocol activity [24].

Time-kill curves evaluated the effect of the selected antibiotics against XDR *P. aeruginosa* isolates. Overall, cefiderocol was the only effective monotherapy across the isolates regardless of their resistance mechanism, achieving a bactericidal effect in six isolates. However, a rapid initial decrease in bacterial load was observed at 8 h, followed by a plateau in seven isolates and final regrowth in the remaining isolate, potentially due to antibiotic degradation or resistance emergence. As these antibiotics are typically administered in 6-8-hour regimens, the results of time-kill curves were assessed at 8 h to better simulate in vivo conditions.

As expected, most MBL- producing isolates behaved similarly to the growth control when exposed to CZA alone. In contrast, aztreonam is susceptible to hydrolysis by SBLs but not by MBLs [36–37], and the expected reduction in bacterial load was observed in MBL-producing strains treated with aztreonam alone. The combination of CZA plus aztreonam has shown activity against MBL- and SBL- producing Gram-negative bacilli, including *P. aeruginosa* isolates [17]. This combination led to a 2–4 log_10_ decrease in bacterial load, achieving bactericidal activity in all but one of the tested isolates at 8 h. As previously noted, aztreonam’s activity in *P. aeruginosa* is compromised by the MexAB-OprM efflux pump [13]. MIC values for aztreonam combined with avibactam indicated that adding avibactam did not significantly reduce aztreonam MICs, with only minor effects observed in certain GES-producing strains.

The chemostat model confirmed that CZA and aztreonam monotherapies lacked bactericidal effects in all isolates. Both cefiderocol and the combination of CZA plus aztreonam achieved sustained bactericidal effects in these isolates, regardless of their resistance mechanism. The significant bacterial reductions observed indicate the potential clinical utility of cefiderocol and CZA plus aztreonam in treating *resistant P. aeruginosa* infections. These findings support further clinical investigation to optimize these regimens for real-world applications in infections with limited therapeutic options.

Understanding these in vitro results provides valuable insights for clinical practice, especially in guiding therapeutic choices for infections with few viable treatment options. Despite limited current clinical data, increasing evidence supports the successful use of cefiderocol in this type of infection [33, 34, 35, 38]. IDSA guidelines currently recommend cefiderocol as the preferred treatment for MBL-producing *P. aeruginosa* infections and as an alternative for difficult-to-treat *P. aeruginosa* infections beyond urinary tract infections [39]. Our findings support cefiderocol’s role not only against MBL-producing isolates but also against GES-carbapenemase-producing isolates. In agreement with our results, a recent in vivo study reported the efficacy of cefiderocol against GES-carbapenemase-producing *P. aeruginosa* in a murine infection model [40]. However, in circumstances where cefiderocol is unavailable, the combination of CZA plus aztreonam may be a suitable alternative, as it demonstrated bactericidal activity in all analyzed isolates and did not show statistically significant differences compared to cefiderocol.

Our study had some limitations. The static antibiotic concentration used in 24-hour time-kill curves limits the direct application of these results to in vivo conditions. A larger number of isolates with varying resistance mechanisms should also be tested. Finally, all isolates exhibited low MICs for cefiderocol compared to the MICs of other antibiotics. However, as cefiderocol is a new antibiotic and may not be accessible in some hospitals, it is crucial to identify alternative therapies for these strains when necessary. The chemostat model used here provided an advantage by simulating dynamic PK/PD conditions that better reflect in vivo antibiotic behaviour, adding rigor to the evaluation of bactericidal effects. Strains in this study were selected based on XDR phenotype and β-lactam resistance mechanism diversity, making the susceptibility testing results a key outcome.

In conclusion, this study showed that both cefiderocol and the combination of CZA plus aztreonam are potentially effective therapies against XDR *P. aeruginosa* carrying MBL and/or SBL carbapenemases. As expected, cefiderocol was the only active monotherapy with a consistent bactericidal effect across all isolates. Our findings underscore the importance of careful antibiotic selection for treating XDR *P. aeruginosa* infections caused by diverse carbapenemase-producing strains. Further studies are necessary to explore and optimize these alternative therapies.

## Electronic supplementary material

Below is the link to the electronic supplementary material.

Supplementary Material 1

## Data Availability

No datasets were generated or analysed during the current study.

## References

[1] Prestinaci F, Pezzotti P, Pantosti A (2015) Antimicrobial resistance: a global multifaceted phenomenon. Pathog Glob Health 109:309–318. 10.1179/2047773215Y.0000000030 26343252 10.1179/2047773215Y.0000000030 PMC4768623

[2] Azam MW, Khan AU (2019) Updates on the pathogenicity status of Pseudomonas aeruginosa. Drug Discov. Today 1(24):350–9. 10.1016/j.drudis.2018.07.003 10.1016/j.drudis.2018.07.003 30036575

[3] Gellatly SL, Hancock REW (2013) *Pseudomonas aeruginosa*: new insights into pathogenesis and host defenses. Pathog Dis 67:159–173. 10.1111/2049-632X.12033 23620179 10.1111/2049-632X.12033

[4] Rodrigo-Troyano A, Melo V, Marcos PJ, Laserna E, Peiro M, Suarez-Cuartin G et al (2018) Pseudomonas aeruginosa in chronic obstructive pulmonary disease patients with frequent hospitalized exacerbations: a prospective multicentre study. Respiration 26:96:417–424. 10.1159/000490190 10.1159/000490190 30041176

[5] del Barrio-Tofiño E, López-Causapé C, Oliver A (2020) Pseudomonas aeruginosa epidemic high-risk clones and their association with horizontally-acquired β-lactamases: 2020 update. Int J Antimicrob Agents 56. 10.1016/j.ijantimicag.2020.106196 10.1016/j.ijantimicag.2020.106196 33045347

[6] Wang MG, Liu ZY, Liao XP, Sun RY, Li RB, Liu Y et al (2021) Retrospective data insight into the global distribution of carbapenemase-producing pseudomonas aeruginosa. Antibiotics 1:10. 10.3390/antibiotics10050548 10.3390/antibiotics10050548 PMC8151531 34065054

[7] Tenover FC, Nicolau DP, Gill CM (2022) Carbapenemase-producing Pseudomonas aeruginosa–an emerging challenge. Emerg Microbes Infect 11:811–814. 10.1080/22221751.2022.2048972 35240944 10.1080/22221751.2022.2048972 PMC8920394

[8] Nordmann P, Poirel L Epidemiology and Diagnostics of Carbapenem Resistance in Gram-negative Bacteria. Clin Infect Dis 2019 12;69:S521–S528. 10.1093/cid/ciz824 10.1093/cid/ciz824 PMC6853758 31724045

[9] Horcajada JP, Montero M, Oliver A, Sorlí L, Luque S, Gómez-Zorrilla S et al (2019) Epidemiology and treatment of multidrug-resistant and extensively drug-resistant Pseudomonas aeruginosa infections. Clin Microbiol Rev 32. 10.1128/CMR.00031-19 10.1128/CMR.00031-19 PMC6730496 31462403

[10] Gordon EM, Duncton MAJ, Wang BJ, Qi L, Fan D, Li X et al (2020) Toward orally absorbed Prodrugs of the Antibiotic Aztreonam. Design of Novel Prodrugs of Sulfate containing drugs. Part 2. ACS Med Chem Lett 11:162–165. 10.1021/acsmedchemlett.9b00534 32071683 10.1021/acsmedchemlett.9b00534 PMC7025374

[11] Ramsey C, MacGowan AP (2016) A review of the pharmacokinetics and pharmacodynamics of aztreonam. J Antimicrob Chemother 71:2704–2712. 10.1093/jac/dkw231 27334663 10.1093/jac/dkw231

[12] Wenzler E, Deraedt MF, Harrington AT, Danizger LH Synergistic activity of ceftazidime-avibactam and aztreonam against serine and metallo-β-lactamase-producing gram-negative pathogens. Diagn Microbiol Infect Dis 2017 1;88:352–354. 10.1016/j.diagmicrobio.2017.05.009 10.1016/j.diagmicrobio.2017.05.009 28602518

[13] Jorth P, McLean K, Ratjen A, Secor PR, Bautista GE, Ravishankar S et al (2017) Evolved aztreonam resistance is multifactorial and can produce hypervirulence in pseudomonas aeruginosa. MBio 8. 10.1128/mBio.00517-17 10.1128/mBio.00517-17 PMC5666152 29089424

[14] Montero MM, Domene Ochoa S, López-Causapé C, Luque S, Sorlí L, Campillo N et al (2021) Time-kill evaluation of antibiotic combinations containing ceftazidime-avibactam against extensively drug-resistant Pseudomonas aeruginosa and their potential role against Ceftazidime-Avibactam-Resistant isolates. Microbiol Spectr 9. 10.1128/spectrum.00585-21 10.1128/spectrum.00585-21 PMC8552783 34319141

[15] Shirley M, Ceftazidime-Avibactam (2018) A review in the treatment of Serious Gram-negative bacterial infections. Drugs 78:675–692. 10.1007/s40265-018-0902-x 29671219 10.1007/s40265-018-0902-x

[16] Wang Y, Wang J, Wang R, Cai Y (2020) Resistance to ceftazidime–avibactam and underlying mechanisms. J Glob Antimicrob Resist 1:22:18–27. 10.1016/j.jgar.2019.12.009 10.1016/j.jgar.2019.12.009 31863899

[17] Lee M, Abbey T, Biagi M, Wenzler E (2021) Activity of aztreonam in combination with ceftazidime–avibactam against serine- and metallo-β-lactamase–producing Pseudomonas aeruginosa. Diagn Microbiol Infect Dis 99. 10.1016/j.diagmicrobio.2020.115227 10.1016/j.diagmicrobio.2020.115227 33086177

[18] Syed YY, Cefiderocol (2021) A review in Serious Gram-negative bacterial infections. Drugs 81:1559–1571. 10.1007/s40265-021-01580-4 34427896 10.1007/s40265-021-01580-4 PMC8383240

[19] Poole K, McKay GA (2003) Iron acquisition and its control in Pseudomonas aeruginosa: many roads lead to Rome. Front Biosci 8. 10.2741/1051 10.2741/1051 12700066

[20] Ito-Horiyama T, Ishii Y, Ito A, Sato T, Nakamura R, Fukuhara N et al (2016) Stability of novel siderophore cephalosporin S-649266 against clinically relevant carbapenemases. Antimicrob Agents Chemother 160:4384–4386. 10.1128/AAC.03098-15 10.1128/AAC.03098-15 PMC4914688 27139465

[21] del Barrio-Tofiño E, López-Causapé C, Cabot G, Rivera A, Benito N, Segura C et al (2017) Genomics and susceptibility profiles of extensively drug-resistant Pseudomonas aeruginosa isolates from Spain. Antimicrob Agents Chemother 61:e01589–e01517. 10.1128/AAC.01589-17 28874376 10.1128/AAC.01589-17 PMC5655108

[22] Del Barrio-Tofinõ E, Zamorano L, Cortes-Lara S, López-Causape C, Sánchez-Diener I, Cabot G et al (2019) Spanish nationwide survey on Pseudomonas aeruginosa antimicrobial resistance mechanisms and epidemiology. J Antimicrob Chemother 74:1825–1835. 10.1093/jac/dkz147 30989186 10.1093/jac/dkz147

[23] Committee E (2022) EUCAST: Clinical breakpoints and dosing of antibiotics [Internet]. Eur. Comm. Antimicrob. Susceptibility Test. [cited 2023 5] https://www.eucast.org/clinical_breakpoints

[24] Hackel MA, Tsuji M, Yamano Y, Echols R, Karlowsky JA, Sahm DF (2019) Reproducibility of broth microdilution MICs for the novel siderophore cephalosporin, cefiderocol, determined using iron-depleted cation-adjusted Mueller-Hinton broth. Diagn Microbiol Infect Dis 94:321–325. 10.1016/j.diagmicrobio.2019.03.003 31029489 10.1016/j.diagmicrobio.2019.03.003

[25] Smith PF, Ballow CH, Booker BM, Forrest A, Schentag JJ (2001) Pharmacokinetics and pharmacodynamics of aztreonam and tobramycin in hospitalized patients. Clin Ther 23:1231–1244 11558860 10.1016/s0149-2918(01)80103-x

[26] Das S, Zhou D, Nichols WW, Townsend A, Newell P, Li J (2020) Selecting the dosage of ceftazidime–avibactam in the perfect storm of nosocomial pneumonia. Eur J Clin Pharmacol 1:76:349. 10.1007/S00228-019-02804-Z 10.1007/s00228-019-02804-z PMC7223046 31836928

[27] Pavithra JYW, Jason S, Pogue M, Wu JY, Srinivas ÁP, Pogue JM Cefiderocol: a Novel Agent for the management of Multidrug-Resistant Gram-negative organisms. 10.6084/m9.figshare.11792034 10.1007/s40121-020-00286-6 PMC7054475 32072491

[28] Lim T-P, Cai Y, Hong Y, Chan ECY, Suranthran S, Teo JQ-M et al (2015) In vitro pharmacodynamics of various antibiotics in combination against extensively drug-resistant Klebsiella pneumoniae. Antimicrob Agents Chemother 59:2515–2524. 10.1128/AAC.03639-14 25691628 10.1128/AAC.03639-14 PMC4394811

[29] Singh R, Kim A, Angela Tanudra M, Harris JJ, McLaughlin RE, Patey S et al (2015) Pharmacokinetics/pharmacodynamics of a β-lactam and β-lactamase inhibitor combination: a novel approach for aztreonam/avibactam. J Antimicrob Chemother 70:2618–2626. 10.1093/jac/dkv132 26024868 10.1093/jac/dkv132

[30] Weber C, Schultze T, Göttig S, Kessel J, Schröder A, Tietgen M et al (2022) Antimicrobial activity of Ceftolozane-Tazobactam, Ceftazidime-Avibactam, and Cefiderocol against Multidrug-Resistant Pseudomonas aeruginosa recovered at a German University Hospital. Microbiol Spectr 2610. 10.1128/spectrum.01697-22 10.1128/spectrum.01697-22 PMC9603231 36190424

[31] Oueslati S, Bogaerts P, Dortet L, Bernabeu S, Ben Lakhal H, Longshaw C et al (2022) In vitro Activity of Cefiderocol and Comparators against Carbapenem-Resistant Gram-Negative Pathogens from France and Belgium. Antibiotics 1:11. 10.3390/antibiotics11101352 10.3390/antibiotics11101352 PMC9598183 36290010

[32] Bassetti M, Echols R, Matsunaga Y, Ariyasu M, Doi Y, Ferrer R et al Efficacy and safety of cefiderocol or best available therapy for the treatment of serious infections caused by carbapenem-resistant Gram-negative bacteria (CREDIBLE-CR): a randomised, open-label, multicentre, pathogen-focused, descriptive, phase 3 trial. Lancet Infect Dis 2021 1;21:226–240. 10.1016/S1473-3099(20)30796-9 10.1016/S1473-3099(20)30796-9 33058795

[33] Viale P, Sandrock CE, Ramirez P, Rossolini GM, Lodise TP (2023) Treatment of critically ill patients with cefiderocol for infections caused by multidrug-resistant pathogens: review of the evidence. Ann Intensive Care 113. 10.1186/s13613-023-01146-5 10.1186/s13613-023-01146-5 PMC10272070 37322293

[34] Meschiari M, Volpi S, Faltoni M, Dolci G, Orlando G, Franceschini E et al (2021) Real-life experience with compassionate use of cefiderocol for difficult-to-treat resistant Pseudomonas aeruginosa (DTR-P) infections. JAC-Antimicrobial Resist 1:3. 10.1093/jacamr/dlab188 10.1093/jacamr/dlab188 PMC8665210 34909691

[35] Fendian ÁM, Albanell-Fernández M, Tuset M, Pitart C, Castro P, Soy D et al (2023) Real-Life Data on the effectiveness and safety of Cefiderocol in severely infected patients: a Case Series. Infect Dis Ther 12:1205–1216. 10.1007/s40121-023-00776-3 36943617 10.1007/s40121-023-00776-3 PMC10029777

[36] Morroni G, Bressan R, Fioriti S, D’achille G, Mingoia M, Cirioni O et al (2021) Antimicrobial activity of aztreonam in combination with old and new β-lactamase inhibitors against mbl and esbl co-producing gram-negative clinical isolates: Possible options for the treatment of complicated infections. Antibiotics 1:10. 10.3390/antibiotics10111341 10.3390/antibiotics10111341 PMC8615000 34827279

[37] Naas T, Poirel L, Nordmann P (2008) Minor extended-spectrum β-lactamases. Clin Microbiol Infect 14:42–52. 10.1111/j.1469-0691.2007.01861.x 18154527 10.1111/j.1469-0691.2007.01861.x

[38] Wicky PH, Poiraud J, Alves M, Patrier J, D’Humières C, Lê M et al Cefiderocol Treatment for Severe Infections due to difficult-to-treat-resistant non-fermentative gram-negative Bacilli in ICU patients: a Case Series and Narrative Literature Review. Antibiot 2023 1;12:991. 10.3390/antibiotics12060991 10.3390/antibiotics12060991 PMC10295316 37370310

[39] Tamma PD, Aitken SL, Bonomo RA, Mathers AJ, van Duin D, Clancy CJ (2023) Infectious Diseases Society of America 2023 Guidance on the Treatment of Antimicrobial Resistant Gram-Negative Infections. Clin. Infect. Dis 18. 10.1093/cid/ciad428 10.1093/cid/ciad428 37463564

[40] Abouelhassan Y, Gill CM, Nicolau DP (2023) Assessing the in vivo efficacy of rational antibiotics and combinations against difficult-to-treat Pseudomonas aeruginosa producing GES β-lactamases. J Antimicrob Chemother 178:1843–1847. 10.1093/jac/dkad098 10.1093/jac/dkad098 PMC10393871 37357368

