# Supplementary material for: Addressing carbapenemase-producing extensively drug-resistant Pseudomonas aeruginosa: the potential of cefiderocol and ceftazidime/avibactam plus aztreonam therapy
Source: Eur J Clin Microbiol Infect Dis. 2025 Feb 18;44(5):1077–87. doi: 10.1007/s10096-025-05061-4 (PMC12062188; doi:10.1007/s10096-025-05061-4)

Supplementary Infomation

Online Resource: Bacterial load over 24 h time-kill experimentsagainst the selected *Pseudomonas aeruginos* isolates.

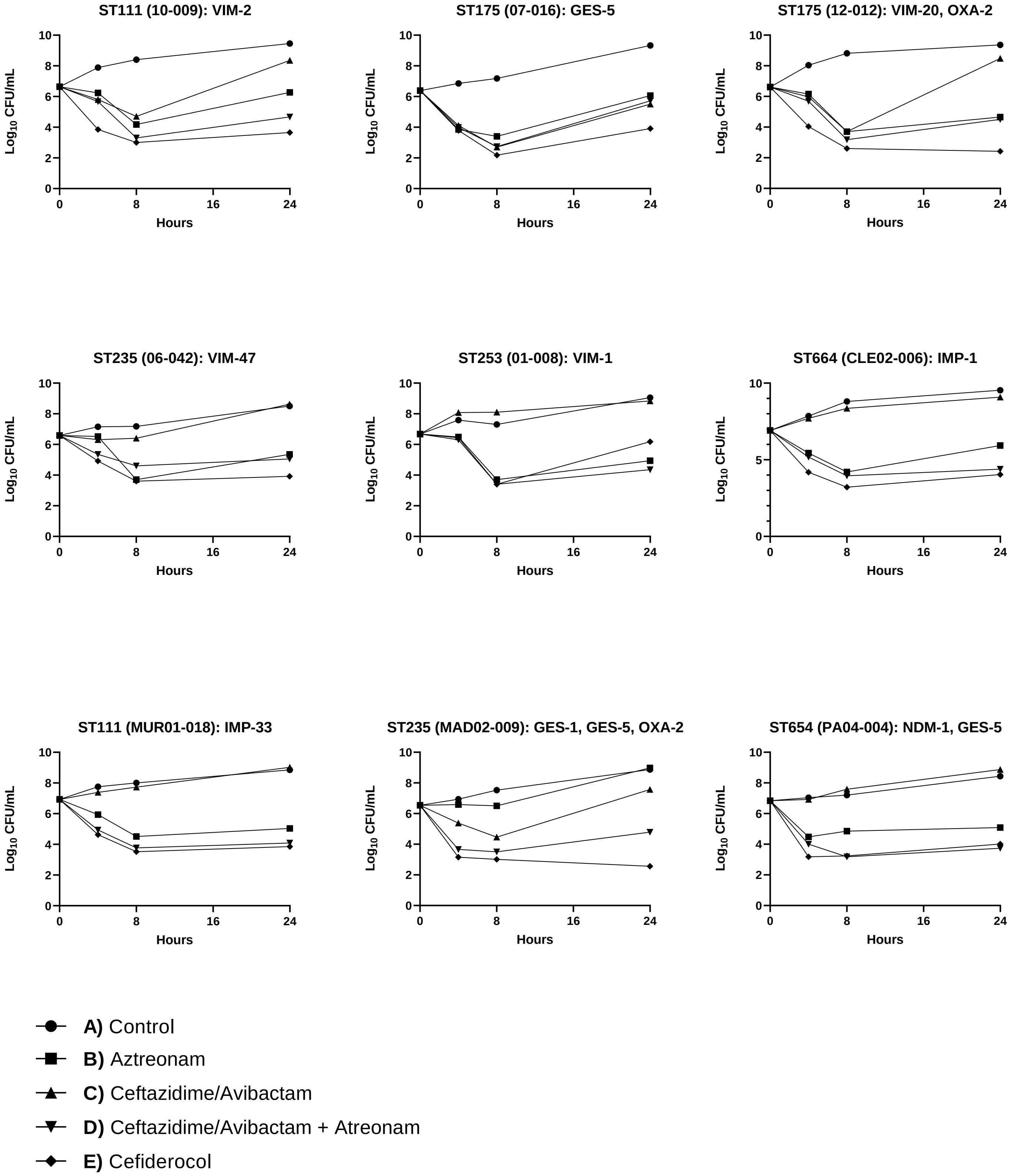

Supplement: Supplementary file 1 — Supplementary Material 1 [file 10096_2025_5061_MOESM1_ESM.pdf]
